# Physical activity and its correlates among school teachers in a semi-urban district of Nepal

**DOI:** 10.1371/journal.pgph.0002000

**Published:** 2023-10-23

**Authors:** Rajan Shrestha, Durga Prasad Pahari, Santoshi Adhikari, Bijay Khatri, Sangita Majhi, Tara Ballav Adhikari, Dinesh Neupane, Per Kallestrup, Abhinav Vaidya

**Affiliations:** 1 Central Department of Public Health, Institute of Medicine, Tribhuvan University, Kathmandu, Nepal; 2 Department of Public Health, Aarhus University, Aarhus C, Denmark; 3 Ethical Review, Monitoring and Evaluation Section, Nepal Health Research Council, Kathmandu, Nepal; 4 Academic and Research Department, Hospital for Children Eye ENT and Rehabilitation Services, Bhaktapur, Nepal; 5 COBIN Project, Nepal Development Society, Chitwan, Nepal; 6 Johns Hopkins Bloomberg School of Public Health, Baltimore, MD, United States of America; 7 Center for Global Health, Department of Public Health, Aarhus University, Aarhus C, Denmark; 8 Department of Community Medicine, Kathmandu Medical College, Kathmandu, Nepal; Universiti Malaya, MALAYSIA

## Abstract

Regular physical activity (PA) is one of the effective strategies for mitigating non-communicable diseases, promoting healthy ageing, and preventing premature mortality. In South Asia, up to 34.0% of adults are insufficiently active, and up to 44.1% of adults in Nepal. We sought to assess self-reported PA status and its correlates among teachers in the semi-urban district of Nepal. A cross-sectional descriptive study was conducted among teachers at randomly selected public secondary schools in Bhaktapur, Nepal, from November 2018-April 2019. PA status was assessed in Metabolic Equivalent to task minutes per week using the International Physical Activity Questionnaire (IPAQ)–Long Form. Point estimates and odds ratios were calculated at a 95% confidence interval, and a p-value <0.05 was considered statistically significant. Among the 360 participants, the mean (SD) age was 40.3 (10.2) years, with 52.5% female participation. A low level of PA was seen among 11.9% (95% CI: 8.4–15.2) of teachers, and more than half (56.0%) of the activity was only moderate intensity. Domestic and garden work was the main contributor (43.0%) of total PA, while leisure time was the least (14.0%). Among the socio-demographic factors, only sex was significantly associated (p = 0.005) with PA. Participants living in locations with walkable areas were 3.4 times (95% CI: 1.6–7.3) more likely to be engaged in moderate-to-high level PA than those without. In our study, the point prevalence of insufficient PA among teachers working at public secondary schools was higher than the national point prevalence. PA promotion programs targeting sedentary populations like school teachers should be developed to reduce the point prevalence of insufficient PA.

## Introduction

Regular physical activity (PA) is one of the most effective strategies for reducing non-communicable diseases (NCDs) and biological risk factors like hypertension and obesity, increasing the quality of life and lifespan [1–4]. Physical inactivity is the fourth leading risk, contributing to 9% of global premature mortality with the increased prevalence of NCDs [5]. Adequate PA can help to prevent and manage NCDs, promote healthy ageing, and prevent premature death [6]. PA also has an interrelationship with the physical, mental, and social well-being of people [7].

World Health Organization (WHO) recommends at least 150–300 minutes weekly of moderate-intensity or at least 75–150 minutes weekly of vigorous-intensity PA for health benefits [3]. Globally, one in four adults have PA below this recommendation.(8) In South Asian countries, there are high variations in national level point prevalence of low-level PA [8], ranging from 7.3% in Bhutan to 41.3% in India, with Nepal having 7.4% [9–14]. The national point prevalence of physical inactivity was higher than in previous national surveys done in Nepal [11, 15, 16]. However, up to 44.1% of adults living in urban areas had insufficient PA. Most people with physical inactivity are job holders mainly involved in desk work and are not engaged in moderate or high-level PA [17–19]. Teachers also engage only in low-level PA and spend their working hours sitting, walking or standing, making them vulnerable groups for NCDs [17, 20]. There is a positive association between PA and teachers’ perceived mental, physical and work-related health [21]. Teachers are considered the main facilitators, change agents and role models for PA promotion to school children and society [22–24]; however, their well-being and PA are rarely discussed globally, including in Nepal. [17, 25–28] Only few studies are available on PA among teachers globally, which reported a high point prevalence of physical inactivity [17, 18, 29].

Nepal has also endorsed a multisectoral action plan for the prevention and control of NCDs and set a 10% relative reduction target in the point prevalence of insufficient PA by 2025; however, the PA status of this vulnerable group, covering a large proportion of Nepal’s population, is not yet available [30, 31]. Therefore, this study aimed to explore PA and its correlates among teachers at public secondary schools (grade 1–10) in Bhaktapur district, Nepal.

## Methods

### Ethics statement

Ethical approval was obtained from the Institutional Review Committee of Institute of Medicine, Tribhuvan University (Ref no. 45(6-11.E)2/075/076). Permission was obtained from municipalities and all the school administrations. Written informed consent was obtained from the participants before starting the interview and the participation in the study was voluntary.

#### Study design and setting

A descriptive cross-sectional study was conducted among teachers at public secondary schools in Bhaktapur, Nepal, from November 2018 to April 2019. Public secondary schools are government schools teaching basic level (grades 1–8) as well as secondary level education (grades 9–12), having larger number of teachers than in other schools.(30) Teachers on those schools are mostly middle-aged adults who spent many years on teaching in same school, as they are recruited after passing the Teacher Service Commission examination and are hired in permanent teaching positions at the same school until retirement. They are generally held in high regard and are respected in Nepali society. This respect for teachers is deeply rooted in the cultural and traditional values of the country [32, 33]. Bhaktapur is a district adjoining the capital city, Kathmandu. The district has urban and rural areas and is one of the rapidly urbanizing zones with the influx of people from all over Nepal. With rapid urbanization, the risk factors of NCDs and NCDs are major health issues in Nepal [30, 34].

#### Study population and sampling

**Fig 1:**
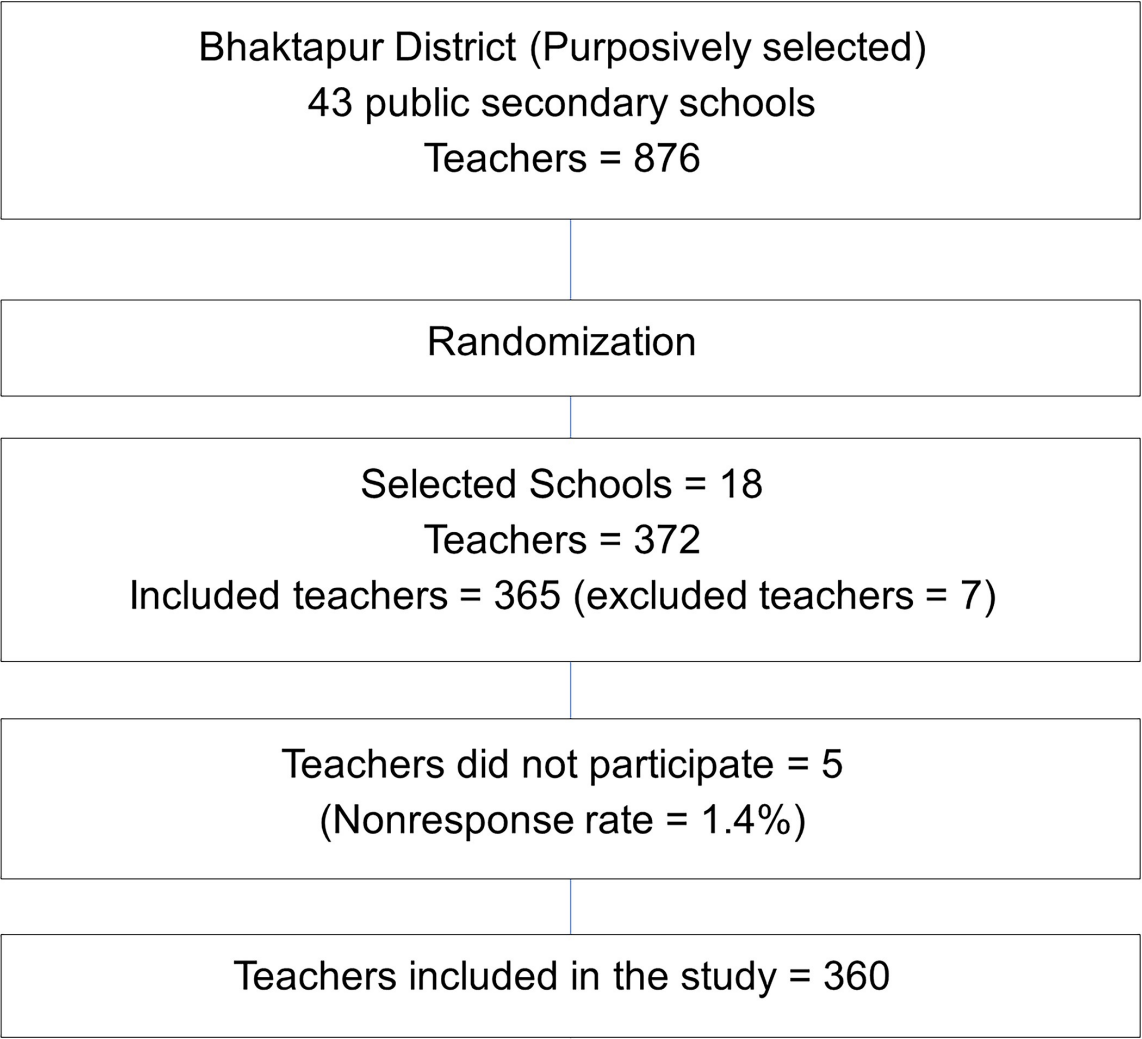
Flow chart showing participant enrollment.

The calculated sample size was 347 using the prevalence study formula (n) = Z^2^ * p (1-p)/e^2^ *1.5 and sample size correction formula as the number of teachers was fixed, n = n/(1+(n-1)/N), where, z = 1.96 at 95% confidence level, p = 0.46 [18], e (allowable error) = 0.05, d (design effect) = 1.5, number of total teachers (N) = 876, and adding of 5% non-response rate [35]. A sampling frame of all public secondary schools in purposively selected Bhaktapur district was prepared. Among the total of 43 public schools, each with 20 teachers on average, 18 schools (347/20 = 17.35≅18) were selected through cluster random sampling, and all teachers from the selected schools were included in the study (S2 Text). Teachers absent for two consecutive days of data collection were excluded from the study. There are no other exclusion criteria used for this study and no drop out in this study. Participants’ enrollment is illustrated in Fig 1.

### Data collection and study tools

We assessed self-reported PA for this study and PA was measured in Metabolic Equivalent to Task (MET)-minutes per week using the International PA Questionnaire (IPAQ)–Long Form through face-to-face interviews. Four domain-specific (leisure time, domestic and garden, work-related, and transport-related), three activity-specific (walking, moderate-intensity, and vigorous-intensity), and total PA MET-minutes/week were calculated as per IPAQ scoring protocol [36]. Moderate level PA was defined as meeting at least three days of vigorous-intensity activity of at least 20 minutes per day or at least five days of moderate-intensity activity and walking of at least 30 minutes per day or at least five days of any combination of walking, moderate-intensity or vigorous-intensity activities achieving a minimum total PA of at least 600 MET-minutes/week. High-level PA was defined as teachers meeting vigorous-intensity activity on at least three days, achieving a minimum total PA of at least 1500 MET-minutes/week, or at least seven days of any combination of walking, moderate-intensity, or vigorous-intensity activities achieving a minimum total PA of at least 3000 MET-minutes/week. Teachers who failed to meet the moderate or high-level criteria were defined as low-level PA. Vigorous Pas take hard physical effort and make the person breathe much harder than usual. In contrast, moderate activities refer to activities that take moderate physical effort and make the person breathe somewhat harder than normal. Similarly, perceived walkable area is considered if the participants have a nearby place where they think they can go for a leisure walk. Other covariates included in this study were–sex, marital status, educational level, level of teaching, and years of teaching experience as socio-demographic variables. Environmental variables included were- availability of PA facilities at home and types (Agricultural land-Teachers having agricultural land are usually involved in ploughing, seeding, maintenance, and harvesting, which could contribute to total PA; kitchen garden, garden, livestock, bicycle, exercise equipment), perceived walkable area near the home, parks, or recreational facilities near home. All these environmental variables were recorded subjectively. Perceived friend’s support, perceived family support, travel time to school, means of transportation, perception of the adequacy of PA, average sitting time, and screen time are also included, which were measured subjectively (S1 Text).

An automated digital blood pressure monitor (OMRON digital gadget) was used to measure blood pressure in the left arm using an adult-sized cuff. Participants were asked to rest for 15 minutes before taking the test, and the average of three systolic and diastolic blood pressure readings was used to calculate the participant’s blood pressure. Height and weight were measured with a portable stadiometer equipped with a digital weighing scale, on a flat surface. Participants were requested to take off their shoes, headgear, and bulky apparel. Participants were instructed to face forward on the weighing scale, look straight ahead, and not tilt. Weight was measured in kilograms, and height was measured in centimeters. Constant tension tape was used to measure waist circumference. At the end of normal expiration, the waist circumference was measured in centimeters on the side of the midpoint between the lower margin of the palpable rib and the top of the iliac crest. BMI was calculated by dividing weight in kilograms by the square of height in meters. Similarly, waist to height ratio (WHtR) is calculated by dividing waist in centimeter by height in centimeter.

### Data management and statistical analysis

Each day, the collected data was checked for completeness, recording errors, and then edited manually on the same day of data collection. Data were entered in EpiData 3.1 data entry format provided with checks to avoid errors in data entry. Data was further cleaned and categorized using MS Excel 365; analysis was done using IBM Statistical Package for Social Sciences (IBM SPSS) 26.0 version. Data were presented in numbers, percentages, mean, and standard deviation. Chi-square tests were performed to examine the differences between categorical variables. We used the logistic regression model to calculate the adjusted odds ratios for groups with low levels and with moderate-to-high level PA entering socio-demographic and enabling variables in the model. Variables with p values of <0.1 in chi-square test, were included in a multivariate logistic regression analysis. The odds ratio was estimated at a 95% confidence interval (CI). A p-value of <0.05 was considered significant.

## Results

### Socio-demographic characteristics

Three hundred sixty teachers aged 18 to 65 years participated in the study with a 1.5% nonresponse rate. The participants’ mean (SD) age was 40.3 (10.2) years. More than half of the participants were female (52.5%). Most participants were involved in teaching basic-level students from grades 1–8 (61.1%). The mean (SD) years of teaching experience was 16.9 (10.2) years (Table 1).

**Table 1:** Socio-demographic characteristics of the participants.

Characteristics		n	%	95% CI
**Sex**	Female	189	52.5	47.3–57.7
	Male	171	47.5	42.3–52.7
**Marital status**	Married	329	91.4	88.5–94.3
	Single/never married	31	8.6	5.7–11.5
**Educational Level**	Higher secondary (Grade 10–12)	75	20.9	16.7–25.1
	University-Bachelor	137	38.1	33.2–43.2
	University-Master and above	147	41.0	35.9–46.1
**Level of teaching**	Basic (Grade 1–8)	220	61.1	56.1–66.1
	Secondary (Grade 9–10)	140	38.9	33.9–43.9
**Years of teaching experience**	Up to 10 years	128	35.5	30.6–40.4
	16–20 years	92	25.5	21.0–30.0
	20 + years	140	38.9	33.9–43.9

### Environmental factors

Among the participants, 235 (65.3%) had facilities to be engaged in PA at home. Most of them had agricultural land (48.3%), while only a few had some exercise equipment (6.9%). Besides that, about 83.6% had nearby walkable areas where they could do morning or evening walks, while only 37.5% of participants had nearby recreational areas (Table 2).

**Table 2:** Characteristics of the participants.

Characteristics		n	%	95% CI
Home PA Facilities	Yes	235	65.3	60.4–70.2
Types of available home PA facilities (Multiple responses)	Agriculture	174	48.3	43.1–53.5
	Kitchen garden	137	38.1	33.1–43.1
	Garden	68	18.9	14.9–22.9
	Livestock	53	14.7	11.0–18.4
	Bicycle	40	11.1	7.9–14.3
	Exercise equipment	25	6.9	4.3–9.5
Walkable area near the home	Yes	301	83.6	79.8–87.4
Parks or recreational facility near home	Yes	135	37.5	32.5–42.5
Travel time to school	Up to 30 min	266	73.9	69.4–78.4
	31–60 min	69	19.2	15.1–23.3
	60+ min	25	6.9	4.3–9.5
Transportation	Walking	131	36.5	31.4–41.4
	Motorcycle	130	36.2	31.1–41.1
	Bus	95	26.5	21.8–31
	Bicycle	3	0.8	-0.1–1.7
Perceived family support	Yes	352	97.8	96.3–99.3
Perceived friends support	Yes	350	97.2	95.5–98.9
Average sitting time per day (minute)	below 158.7	199	55.4	50.2–60.4
	158.7 and above	160	44.6	39.3–49.5
Screen time	Up to 2 hours	210	58.3	53.2–63.4
	More than 2 hours	150	41.7	36.6–46.8
Blood Pressure	SBP < 140 and/or DBP < 90	294	81.7	77.7–85.7
	SBP ≥140 and/or DBP ≥ 90	66	18.3	14.3–22.3
Body Mass Index	< 30	312	86.7	83.2–90.2
	≥ 30	48	13.3	9.8–16.8
Waist to Height Ratio	< 0.6	191	53.1	47.9–58.3
	≥ 0.6	169	46.9	41.7–52.1

Mean (SD) screen time = 2.37 (1.78) hours; Mean (SD) sitting time = 158.74 (88.13) Minutes

### Physical activity

Among the participants, a low level of PA was found in 11.9% (95% CI: 8.6%-15.2%). While moderate and high-level PA was found in 81.4% (95% CI: 77.4%-85.4%) and 6.7% (95% CI:4.1%-9.3%), respectively. WHO’s global recommendation of PA was met by 90.3% (95%CI:87.2%-93.4%) of participants (Table 3).

**Table 3:** Level of PA.

Characteristics	n	Percentage (95% CI)
**IPAQ Classification**		
Low PA	43	11.9 (8.4–15.2)
Moderate PA	293	81.4 (77.4–85.4)
High PA	24	6.7 (4.1–9.3)
Meeting WHO global recommendation on PA	325	90.3 (87.2–93.4)

Mean (SD) of total MET-minutes/week was 3369.4 (2413.0). In terms of activity-specific PA, moderate activity (56.0%) is the most significant contributor to the total MET-minutes of PA accumulated by participants in a week, followed by walking (31.0%) and vigorous activity (13,0%). Home and garden work (43.0%) contributed a more significant proportion of the total MET-minutes/week of the participants, whereas leisure-time activity contributed only 14.0%. Work (21.0%) and Transport (20.0%) domains contributed almost equally to the proportion of the total MET-minutes/week of PA gained by teachers (Table 4).

**Table 4:** Activity and domain-specific PA composition (n = 360).

	Total (%)	Median MET—minutes/week	Interquartile range
**Total PA Score**		2,979.0	3,309.0
**Activity Specific**			
Walking	31.0	792.0	1,266.4
Moderate intensity	56.0	1,452.0	2,486.2
Vigorous intensity	13.0	0	0
**Domain-Specific**			
Work	21.0	0	720.0
Transport	22.0	462.0	1,101.8
Domestic and garden	43.0	1,080.0	2,263.0
Leisure time	14.0	198.0	693.0

### Univariate comparison of active and inactive study participants

Among the socio-demographic factors, only sex was significantly associated (p = 0.005) with PA. Similarly, the availability of walkable areas in the neighborhood (p = 0.009), using bicycles or walking to reach the school (p = 0.019, Cramer’s V = 0.124), and having any facility at home to be physically active (p = 0.002) had a significant association with engaging in moderate to high-level PA (Table 5).

**Table 5:** Socio-demographic factors and level of PA.

	Characteristics	PA Level		P-value
		Moderate to High n (%)	Low n (%)	
All		317 (88.1)	43 (11.9)	
Sex	Female	175 (92.6)	14 (7.4)	0.005
	Male	142 (83.0)	29 (17.0)	
Age	15–29	57 (90.5)	6 (9.5)	0.400
	30–44	128 (85.3)	22 (14.7)	
	45–65	132 (89.8)	15 (10.2)	
Educational level	Up to bachelor	189 (89.2)	23 (10.8)	0.429
	Master and above	127 (86.4)	20 (13.6)	
Teaching years	Up to 10 years	112 (87.5)	16 (12.5)	0.931
	11–20 years	82 (89.1)	10 (10.9)	
	20+ years	123 (87.9)	17 (12.1)	
Marital status	Married	280 (88.3)	37 (11.7)	0.665
	Unmarried	37 (86.0)	6 (14.0)	
Teaching level	Basic level	192 (87.3)	28 (12.7)	0.566
	Secondary level	125 (89.3)	15 (10.7)	
Neighborhood walkable area	Yes	271 (90.0)	30 (10.0)	0.009
	No	46 (78.0)	13 (22.0)	
Mode of Transportation	Walking/ Bicycle	125 (93.3)	9 (6.7)	0.019
	Bus/ Motorbike	192 (85.0)	34 (15.0)	
Availability of recreational park	Yes	109 (86.5)	17 (13.5)	0.506
	No	208 (88.9)	26 (11.1)	
Neighborhood PA facilities	Yes	114 (84.4)	21 (15.6)	0.102
	No	203 (90.2)	22 (9.8)	
Home PA facilities	Yes	216 (91.9)	19 (8.1)	0.002
	No	101 (80.8)	24 (19.2)	
Home to school travel time	≤ 30 mins	229 (86.1)	37 (13.9)	0.053
	>30 mins	88 (93.6)	6 (6.4)	
Family support	Yes	311 (88.4)	41 (11.6)	1.000 ^#^
	No	6 (85.7)	1 (14.3)	
Friends support	Yes	8 (80.0)	2 (20.0)	0.763 ^#^
	No	309 (88.3)	41 (11.7)	
Blood Pressure	SBP < 140 and/or DBP < 90	262 (89.1)	32 (10.9)	0.191
	SBP ≥140 and/or DBP ≥ 90	55 (83.3)	11 (16.7)	
Body Mass Index	< 30	277 (88.8)	35 (11.2)	0.279
	≥ 30	40 (83.3)	8 (16.7)	
Waist to Height Ratio	< 0.6	172 (90.1)	19 (9.9)	0.214
	≥ 0.6	145 (85.8)	24 (14.2)	

# Fisher’s exact test value, and others are chi-square test value

Teachers who had a walkable area near their home were about three times more likely to be engaged in moderate to a high level of PA (AOR = 3.4, 95% CI = 1.6–7.3) than those who did not have one. Similarly, teachers who had facilities for being physically active at their homes were about 2.9 times more likely to be engaged in moderate to high-level PA (AOR 2.9, 95% CI = 1.5–5.8) than those who did not have any. Being female and perception of adequate PA are also significantly associated with PA (Table 6).

**Table 6:** Correlates of engagement in moderate to vigorous PA.

	Characteristics	Adjusted OR* (95% CI)	P-value	Pseudo R Square^#^
Sex (Ref: Male)	Female	2.8 (1.4–5.5)	0.004	
Age		1.0 (0.9–1.0)	0.310	0.047
Availability of walkable area near the home	Yes	3.4 (1.6–7.3)	0.002	0.093
Means of transportation (Ref: bike/bus)	walking/ cycle	2.1 (0.9–4.6)	0.058	0.068
Availability of PA facility at home	Yes	2.9 (1.5–5.8)	0.001	0.102
Blood pressure level (Ref: SBP ≥140 and DBP ≥ 90)	SBP<140 and/or DBP < 90	1.5 (0.7–3.4)	0.293	0.053
Perception of the adequacy of PA	Yes	2.5 (1.2–5.4)	0.013	0.082
Travel time to school (Ref: ≤ 30 mins)	>30 mins	2.0 (0.8–5.1)	0.124	0.061
Body Mass Index (Ref: ≥ 30)	< 30	1.9 (0.8–4.7)	0.131	0.058
Waist to Height Ratio (Ref: ≥ 0.6)	< 0.6	1.9 (0.9–3.9)	0.054	0.067

* Adjusted for age and sex variables using binary logistic regression

^#^ Nagelkerke R Square value

## Discussion

The present study showed a high proportion (90.3%) of participants meeting WHO-recommended PA for health benefits. More than one in ten (11.9%) teachers working at public secondary schools of Bhaktapur have a low level of PA which is higher than the national point prevalence (7.4%) reported in the WHO STEPS survey 2019 [11]. The district included in our study is in Kathmandu Valley, the most developed valley and neighboring the capital city of Nepal, which may be a possible reason for the higher point prevalence of low-level PA than observed in data representing the whole country.

The point prevalence of low-level PA reported in our study is far below the findings from almost all other available literature. In our study, the point prevalence of low-level PA is less than half the point prevalence among general adults (27.5% in 2016 and 31.1% in 2012) globally [37, 38]. The point prevalence is also way below the point prevalence of low-level PA among general population reported from a similar area of the same district (43.4%).(35) Similarly, our finding is lower than neighboring country India’s national point prevalence (41.4%) of low-level PA.(36) We used the IPAQ to assess PA, which evaluates a week’s PA prior to the day of data collection. In contrast, those studies used the Global Physical Activity Questionnaire (GPAQ), which evaluates the average PA performed in a typical week. In addition, another possible reason for the difference in point prevalence may be due to the difference in the study population. Another multi-country study found that regular engagement in moderate PA among the general population was highest for Nepal (69.7%), than India (57.6%), Sri Lanka (49.7%), and Bangladesh (37.4%) [39]. However, these studies are not directly comparable, since they used World Health Survey data where the PA assessment is not based on Metabolic Equivalent to Task.

We used IPAQ because it has a well understandable comprehensive analysis plan and allows us to calculate PA categories–low, moderate and high PA; domain-specific—walking, moderate and vigorous intensity; and domain-specific -work, transport, domestic and garden and leisure time; which is not available in GPAQ, besides the WHO recommendation used as cut off.(32) [40] This tool is comprehensive and reduces recall bias as it is based on activities performed in the last seven days rather than an activity performed in a typical week, as well as questions that are understandable to the participants [41].

The point prevalence of low-level PA reported in our study is far below than activity level of teachers in urban south India (46.5%) [17]. This study only assessed 24 hours PA levels and may missed the PA done on other days, which is covered in our study. There are no other comparable studies done in low- and middle-income countries. However, the point prevalence of insufficient PA is very less than the reported point prevalence in few available studies done in upper middle-income country and high-income-country [29, 42]. A study conducted among teachers at public secondary schools in Sao Paulo, Brazil, reported a higher point prevalence of low-level PA (46.3%) than this study [42]. Similarly, teachers in our study is very less inactive than Croatian teachers (24.0%) [29]. This might be due to the difference in development, high HDI, GDP, and lifestyle differences between these countries and Nepal. Sex is significantly associated with low-level PA in our study (P>0.05) and the study done in Brazil (P>0.01), but the proportion was different. In our study, the proportion of males and females with low-level PA was 17% and 7.4%, respectively, whereas 53.0% and 42.9% in the study done in Brazil [18]. Another study done among people with diabetes in Pokhara and Lalitpur metropolises using GPAQ also demonstrated a higher point prevalence of low-level PA (20.4%) than our study [43].

Our study showed a significant association between PA and factors like sex, walkable area near home, means of transportation, availability of PA facilities at home, perception of the adequacy of PA, and blood pressure level before adjustment, while having PA facilities at home and having a walkable area near home were only significant after adjustment. Moderate to high PA was higher in females than males, which contrasts with the study of PA in the world population, as well as Croatian, South Indian and Brazilian teachers, where females are more inactive than men [17, 18, 38, 42]. This may be because females have to do household chores solely, even when they are engaged in jobs, which is not the same for males in the Nepalese society. Even though, leisure time PA is associated more with positive perceived wellbeing, it is found to be the least contributor of PA among teachers in our study, which is also consistent with the findings among Brazilian teachers, as well as civil servants and general population in Nepal [19, 42, 44]. Availability of walkable areas near home was significantly associated with involvement in moderate to high levels of PA (p<0.05), which is also supported by a study done among older adults in Australia [45]. This might be due to the 31% contribution by walking in total PA. Another factor significantly associated with a moderate to a high level of PA was the availability of PA facilities at home. This PA is also consistent with the findings of a study done among residents in San Diego, California [46], and another study done among students of San Diego State University, California [47].

The strength of our study lies in highlighting the burden of insufficient PA among sedentary populations like school teachers from low- and middle-income countries. However, this study has several limitations to report. PA is based on subjective assessment and may be influenced by self-reported miscalculations. The tool only assesses the PA done for a week, potentially missing the activities done before and after a week of data collection days or just done on those weeks that were not done normally. There could have been recall bias as the participants should recall different activities they had done in a week [48, 49].

## Conclusion

The study concludes that insufficient PA among school teachers is higher than the national point prevalence, with very less leisure time PA. Being female, the availability of walkable areas near homes and PA facilities in the neighborhood were enabling factors of PA. PA promotion programs targeting sedentary populations like school teachers should be developed to reduce the point prevalence of insufficient PA. Large-scale community-based cohort studies using objective measurements of PA should be carried out to find the actual status of PA.

## Supporting information

S1 Checklist STROBE statement. (DOC)

S1 Data Full study dataset. (CSV)

S1 Text Characteristics. (DOCX)

S2 Text Sample size calculation. (DOCX)
