# Supplementary material for: Physical activity and its correlates among school teachers in a semi-urban district of Nepal
Source: PLOS Glob Public Health. 2023 Oct 23;3(10):e0002000. doi: 10.1371/journal.pgph.0002000 (PMC10593206; doi:10.1371/journal.pgph.0002000)
Supplement: S1 Text — (DOCX) [file pgph.0002000.s003.docx]

| **Characteristics** | **Items included** |
| --- | --- |
| Walkable area near the home | Yes/No –  Availability of perceived area nearby, where participants think they can go for leisure time walking (morning/evening walk) if they want |
| Perceived family support | Yes/No –  perceived support from family if participants want to engage in physical activity |
| Perceived friends support | Yes/No –  perceived support from close friends if participants want to engage in physical activity |
| Average sitting time per day (minute) | Subjectively reported average sitting time in minutes |
| Screen time | Subjectively reported average screen used time (all types of screen) |

Supplementary File
