# Supplementary material for: Physical activity and its correlates among school teachers in a semi-urban district of Nepal
Source: PLOS Glob Public Health. 2023 Oct 23;3(10):e0002000. doi: 10.1371/journal.pgph.0002000 (PMC10593206; doi:10.1371/journal.pgph.0002000)
Supplement: S2 Text — (DOCX) [file pgph.0002000.s004.docx]

The sample size was calculated by using the following formula ([15](#_ENREF_15))

Sample size (n) = $\frac{z^{2}pq}{e^{2}}$ ×1.5 (design effect)

Where, z=1.96 at 95% confidence level,

p = 0.46 ([31](#_ENREF_31)), q = 1-p = 0.54

e (allowable error) = 0.05

d (design effect) =1.5

Calculated sample size, n = 573,

Since, the sample population is (finite), Total number of teachers in the study area (N)= 876


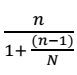


Sample size with correction =

So, sample size = 347,

with 5% non-response rate, Final sample size = 364
